# Supplementary material for: Borrelia burgdorferi lacking all cp32 prophage plasmids retains full infectivity in mice
Source: EMBO Rep. 2025 Mar 19;26(8):1997–2012. doi: 10.1038/s44319-025-00378-9 (PMC12018966; doi:10.1038/s44319-025-00378-9)
Supplement: Supplementary file 6 — Expanded View Figures [file 44319_2025_378_MOESM6_ESM.pdf]

Expanded View Figures

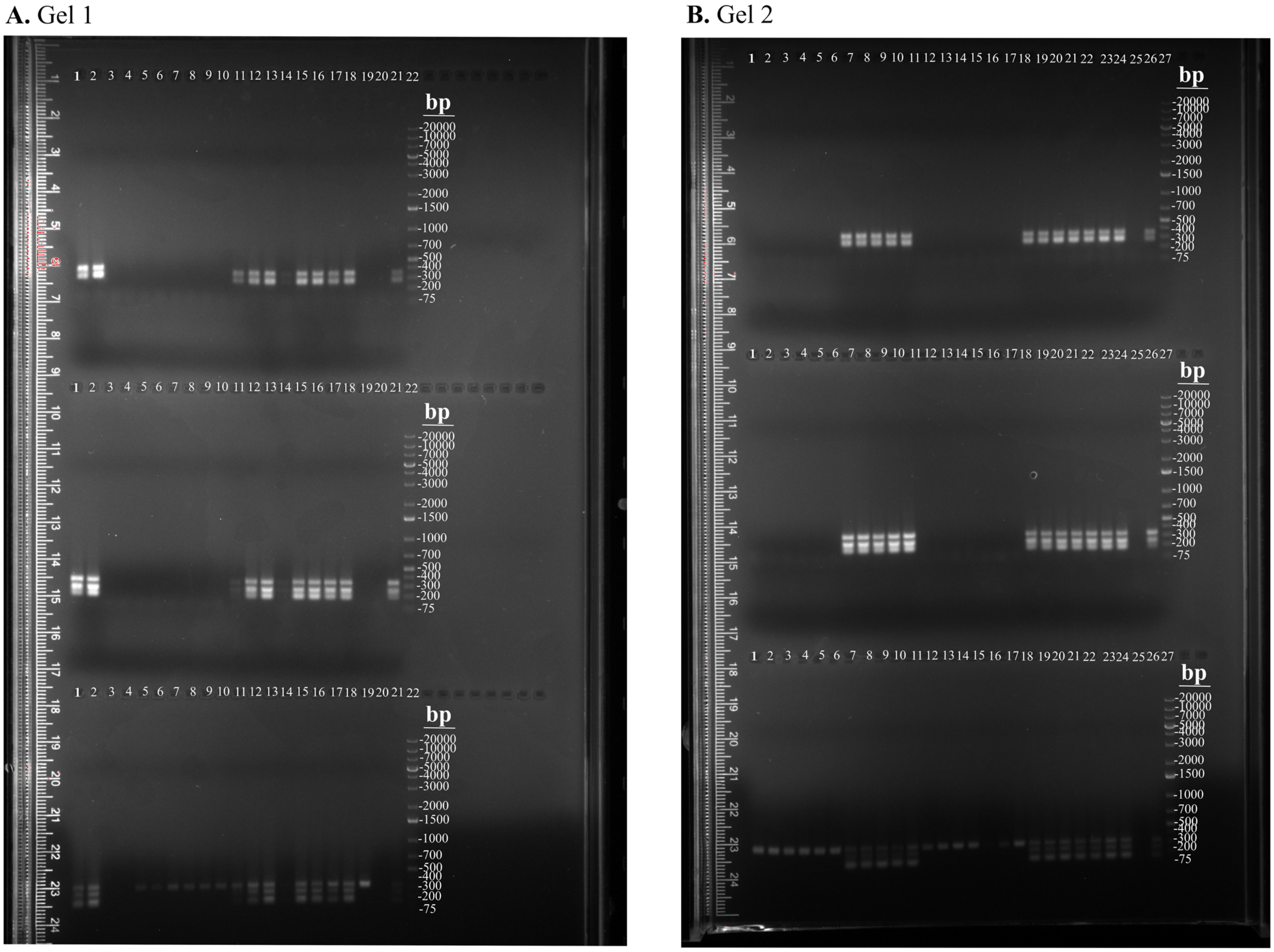

Figure EV1. Multiplex PCR of murine ear outgrowths.

To ensure that mice were infected with the correct *B. burgdorferi* derivative, outgrowths from ear tissues were analyzed by multiplex PCR using the circular plasmid primer sets. (**A**) Gel 1 was loaded as follows: 1. mouse 23-B (wt, 5 weeks); 2. mouse 134-N (wt, 4 weeks); 3. mouse 23-L (uninfected, 5 weeks); 4. mouse 23-R (uninfected, 5 weeks); 5. mouse 24-B (Δcp32, 5 weeks); 6. mouse 24-L (Δcp32, 5 weeks); 7. mouse 24-R (Δcp32, 5 weeks); 8. mouse 25-B (Δcp32, 5 weeks); 9. mouse 25-L (Δcp32, 5 weeks); 10. mouse 25-R (Δcp32, 5 weeks); 11. mouse 25-2 R (Δcp32, 5 weeks); 12. mouse 130-B (wt, 2 weeks); 13. mouse 130-L (wt, 2 weeks); 14. mouse 130-N (wt, 2 weeks); 15. mouse 130-R (wt, 2 weeks); 16. mouse 131-L (wt, 2 weeks); 17. mouse 131-N (wt, 2 weeks); 18. mouse 131-R (wt, 2 weeks); 19. mouse 132-L (Δcp32, 2 weeks); 20. Negative PCR control (water); 21. Positive PCR control (B31-A3); 22. Ladder (GeneRuler 1 kb plus). (**B**) Gel 2 was loaded as follows: 1. mouse 18-L (Δcp32, 2 weeks); 2. mouse 18-N (Δcp32, 2 weeks); 3. mouse 18-R (Δcp32, 2 weeks); 4. mouse 19-B (Δcp32, 2 weeks); 5. mouse 19-L (Δcp32, 2 weeks); 6. mouse 19-N (Δcp32, 2 weeks); 7. mouse 133-L (wt, 4 weeks); 8. mouse 133-N (wt, 4 weeks); 9. mouse 133-R (wt, 4 weeks); 10. mouse 134-L (wt, 4 weeks); 11. mouse 134-R (wt, 4 weeks); 12. mouse 21-L (Δcp32, 4 weeks); 13. mouse 21-N (Δcp32, 4 weeks); 14. mouse 21-R (Δcp32, 4 weeks); 15. mouse 22-B (Δcp32, 4 weeks); 16. mouse 22-L (Δcp32, 4 weeks); 17. mouse 22-N (Δcp32, 4 weeks); 18. mouse 22-R (Δcp32, 4 weeks); 19. mouse 136-B (wt, 5 weeks); 20. mouse 136-N (wt, 5 weeks); 21. mouse 136-R (wt, 5 weeks); 22. mouse 137-L (wt, 5 weeks); 23. mouse 137-N (wt, 5 weeks); 24. mouse 137-R (wt, 5 weeks); 25. Negative PCR control (water); 26. Positive PCR control (B31-A3); and 27. Ladder (GeneRuler 1 kb plus). Both (**A**) and (**B**) were loaded so that primer mix cp1 (amplifying cp32-8 at 375 bp, cp32-4 at 300 bp, and cp9 at 226 bp), cp2 (amplifying cp32-1 at 350 bp, cp32-6 at 276 bp, and cp32-7 at 200 bp), and cp3 (amplifying cp26 at 325 bp, cp32-9 at 250 bp, and cp32-3 at 170 bp) are represented by the top, middle, and bottom wells, respectively.

© The Author(s)

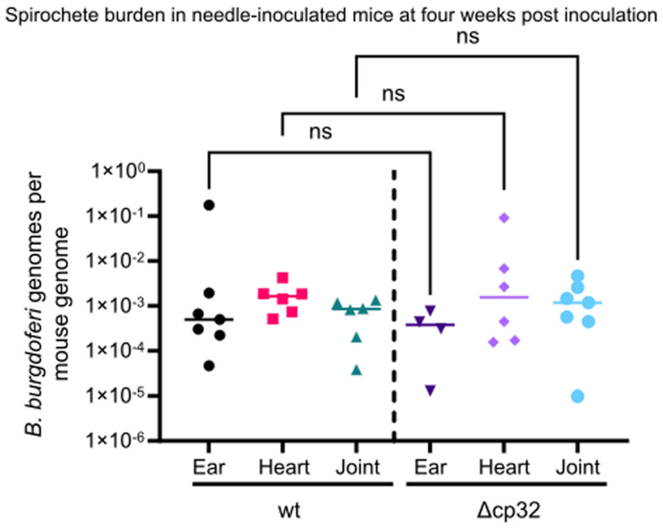

Strain	Tissue	Number of tissues analyzed	Time point of tissue collection
wt	Ear	6	4 weeks
wt	Heart	6	4 weeks
wt	Joint	4	4 weeks
Δcp32	Ear	4	4 weeks
Δcp32	Heart	6	4 weeks
Δcp32	Joint	7	4 weeks

Figure EV2. Infectivity of wt and Δcp32 spirochetes in mice at 4-weeks post injection.

Similar numbers of wt and Δcp32 genomes were detected by qPCR in ear, heart, and joint tissues of needle inoculated mice at 4-weeks post-injection. The number of tissues that were analyzed and the time points of tissue collection are shown as a table beneath the graph. Mann–Whitney nonparametric test was used to determine significance. Data are presented in graphs as individual values of biological replicates, with the mean value indicated. ****p*-value < 0.0005. Source data are available online for this figure.

 © The Author(s)
